# Browning of the white adipose tissue regulation: new insights into nutritional and metabolic relevance in health and diseases

**DOI:** 10.1186/s12986-022-00694-0

**Published:** 2022-09-06

**Authors:** Sabrina Azevedo Machado, Gabriel Pasquarelli-do-Nascimento, Debora Santos da Silva, Gabriel Ribeiro Farias, Igor de Oliveira Santos, Luana Borges Baptista, Kelly Grace Magalhães

**Affiliations:** Laboratory of Immunology and Inflammation, Department of Cell Biology, University of Brasilia, Brasília, DF Brazil

**Keywords:** White adipose tissue, Browning, UCP1, Life-style

## Abstract

Adipose tissues are dynamic tissues that play crucial physiological roles in maintaining health and homeostasis. Although white adipose tissue and brown adipose tissue are currently considered key endocrine organs, they differ functionally and morphologically. The existence of the beige or brite adipocytes, cells displaying intermediary characteristics between white and brown adipocytes, illustrates the plastic nature of the adipose tissue. These cells are generated through white adipose tissue browning, a process associated with augmented non-shivering thermogenesis and metabolic capacity. This process involves the upregulation of the uncoupling protein 1, a molecule that uncouples the respiratory chain from Adenosine triphosphate synthesis, producing heat. β-3 adrenergic receptor system is one important mediator of white adipose tissue browning, during cold exposure. Surprisingly, hyperthermia may also induce beige activation and white adipose tissue beiging. Physical exercising copes with increased levels of specific molecules, including Beta-Aminoisobutyric acid, irisin, and Fibroblast growth factor 21 (FGF21), which induce adipose tissue browning. FGF21 is a stress-responsive hormone that interacts with beta-klotho. The central roles played by hormones in the browning process highlight the relevance of the individual lifestyle, including circadian rhythm and diet. Circadian rhythm involves the sleep–wake cycle and is regulated by melatonin, a hormone associated with UCP1 level upregulation. In contrast to the pro-inflammatory and adipose tissue disrupting effects of the western diet, specific food items, including capsaicin and n-3 polyunsaturated fatty acids, and dietary interventions such as calorie restriction and intermittent fasting, favor white adipose tissue browning and metabolic efficiency. The intestinal microbiome has also been pictured as a key factor in regulating white tissue browning, as it modulates bile acid levels, important molecules for the thermogenic program activation. During embryogenesis, in which adipose tissue formation is affected by Bone morphogenetic proteins that regulate gene expression, the stimuli herein discussed influence an orchestra of gene expression regulators, including a plethora of transcription factors, and chromatin remodeling enzymes, and non-coding RNAs. Considering the detrimental effects of adipose tissue browning and the disparities between adipose tissue characteristics in mice and humans, further efforts will benefit a better understanding of adipose tissue plasticity biology and its applicability to managing the overwhelming burden of several chronic diseases.

## Background

The adipose tissues (ATs) are endocrine and dynamic organs that display high morphological and functional plasticity. White AT (WAT) was named that way because it presents white adipocytes in its composition. In contrast, brown AT (BAT) has as its main integrant the brown adipocytes [1]. Both ATs play various physiological roles, including energy storage, endocrine regulation, and thermogenesis. As a means of adapting, mammalians developed a mechanism to maintain their body temperatures under unfavorable climates [2]. This process, called adaptative thermogenesis, occurs due to the elevated plasticity of ATs, which allows reversible changes in their morphology and functions [3]. Studies have shown the capacity of progenitor cells as well as mature adipocytes to differentiate into a model that presents similarities with the brown profile, called brite or beige AT [4–6]. When this phenomenon occurs in mature adipocytes it is called browning of WAT [4].

**Fig. 1:**
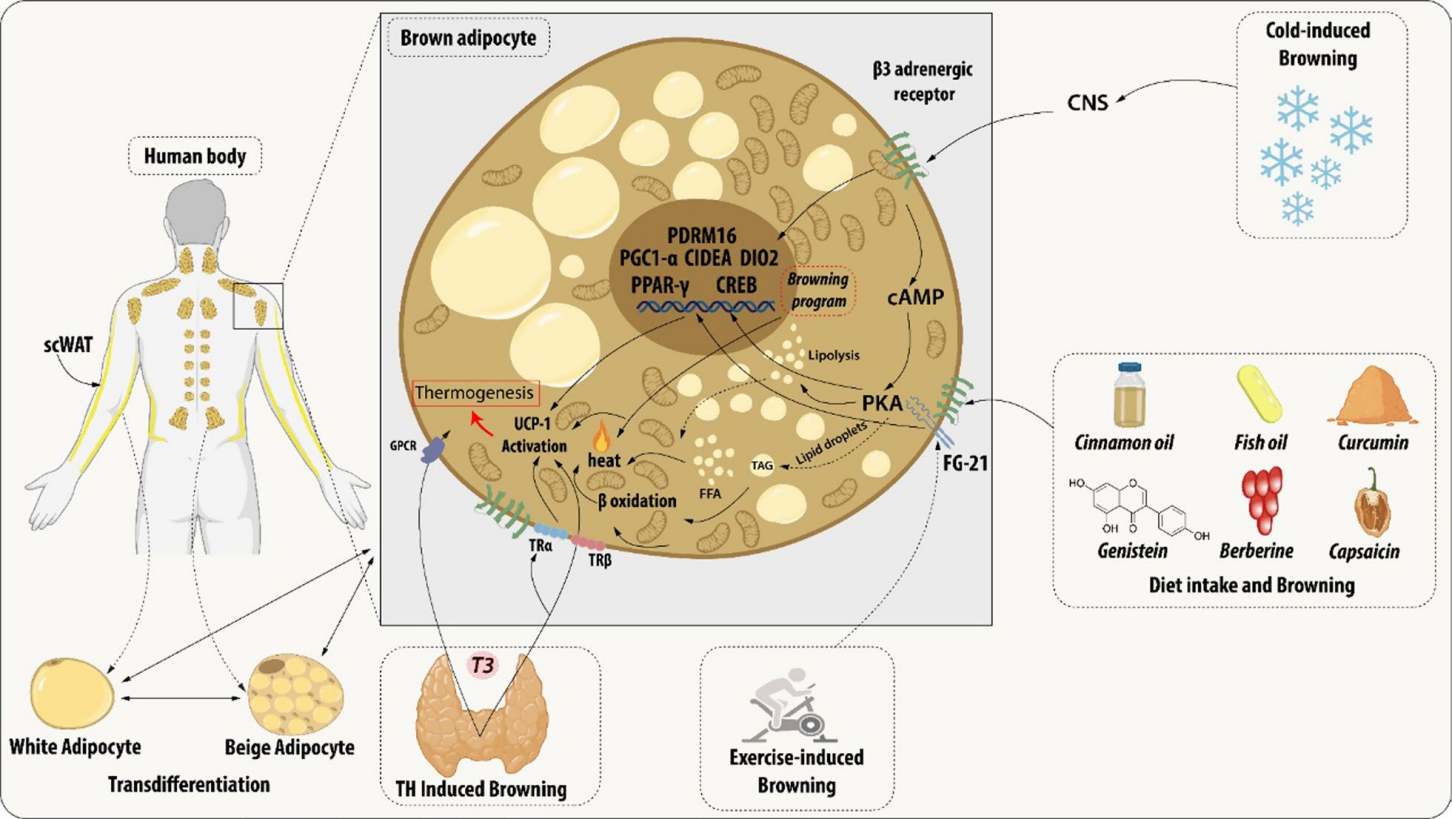
Brown adipocyte regulation by exogenous agents. Thyroid hormones act on thermogenesis through interaction with their Thyroid receptors (TR) and the G-protein-coupled receptor (GPCR). TRα promotes an increase in adrenergic signaling while TRβ acts to stimulate uncoupling protein 1 (UCP1). β3 receptor (β3-AR) is expressed constitutively on the surface of the adipocyte and acts to regulate the transcription and activation of genes related to mitochondrial biogenesis, brown adipocyte differentiation, and lipid storage. This receptor can be activated through cold, which is the main mechanism for activating browning, agonist drugs, or diet. Chronic exposure to cold and food intake, such as curcumin and fish oil, promotes thermogenesis by releasing catecholamines from the central nervous system (CNS) that bind to β-AR, thus initiating a signaling cascade. An increase in the concentration of cAMP is elicited which consequently leads to the enhanced activity of protein kinase A (PKA) which promotes the cAMP-response element-binding protein (CREB). This pathway is related to the transcription of thermogenic genes such as peroxisome proliferator-activated receptor γ (PPARγ), Type II iodothyronine deiodinase (DIO2), PR domain containing 16 (PRDM16), Peroxisome proliferator-activated receptor gamma coactivator 1-alpha (PGC-1α) and Cell death-inducing DNA fragmentation factor-like effector A (CIDEA). The other dietary components can participate in the induction of browning through the modulation of the gut microbiota promoting the increase of the expression of thermogenic genes. The release of growth factor 21 (FGF21) is mediated by the practice of physical exercises and physiological changes. FGF21 will interact with its FGFR receptor and that activation induces a self-phosphorylation of the FGFR that mediates the activation of pathways related to increased expression of UCP1

During the browning process, WAT show increased mitochondrial number, augmented energy expenditure, fat multilocularization, and thermogenic genes expressions, such as Peroxisome proliferator-activated receptor-a (PPARα) and PPARγ, PR domain containing 16 (PRDM16), peroxisome proliferator-activated receptor-gamma coactivator 1 a (PGC1-α), cell death-inducing DNA fragmentation factor-like effector A (CIDEA), and Uncoupling Protein 1 (UCP1) [7–9]. WAT browning occurs in specific conditions through exposure to certain stimuli such as cold, thyroid hormones, diet, natriuretic peptides, medication, and exercise [10–14] (Fig. 1).

The increase in energy expenditure can act as a therapeutic approach to metabolic syndrome, and also can be associated with poor prognosis of diseases associated with hypermetabolism [15–18]. For this reason, efforts have been employed to identify the key participants in the regulation of browning. This review aims to present and describe the current studies related to both endogenous and exogenous most relevant agents and their biological mechanisms at biochemical and molecular levels.

## Introduction

Originally cited as energy storage organs, exclusively, ATs are currently known to express and secrete a variety of bioactive peptides, the adipokines, including leptin, resistin, vaspin, visfatin, hepcidin, adiponectin, and inflammatory cytokines. These bioactive secreted factors act both locally and systematically, modulating different biological processes and consequently influencing the metabolism of various organs, such as the liver, muscle, pancreas, and brain via endocrine mechanisms [19, 20]. Besides adipocytes, AT contains an extracellular matrix, nerve tissue, stromovascular cells, and immune cells, which together act as an integrated unit [21].

Presently, two main subtypes of ATs have been described: WAT and BAT. Brown and white adipocytes have widely different morphologies, not only in terms of composition but also in the form of lipid storage (number and size of lipid droplets) and the disposition and number of mitochondria. These differences correspond to distinct functional roles, diverging in energy metabolism, storage, and distribution [19, 22].

WAT, the most abundant AT in the body, contains the white adipocytes, which present unilocular lipid droplets, scarce mitochondria, and lipid storage capacity [23]. Since the discovery of the adipokines, WAT is also recognized as an important endocrine organ, actively participating in the regulation of physiologic and pathologic processes, including immunity and inflammation [24, 25]. Widely distributed throughout the body, there are two main representative types of WATs, the visceral WAT (vWAT) and the subcutaneous WAT (scWAT). While one is distributed around organs and provides protective padding, the other is located under the skin and provides insulation against heat or cold, respectively [26].

In contrast, brown adipocytes display multilocular lipid droplets, a large number of mitochondria, and thermogenic capacity due to elevated uncoupling protein 1 (UCP1) amounts anchored in its mitochondrial inner membrane [27]. The BAT utilizes this high mitochondrial content and elevated UCP1 amounts to uncouple oxidative phosphorylation from adenosine triphosphate (ATP) synthesis to dissipate chemical energy as heat [28]. Thus, BAT affects the metabolism of the entire body, being able to alter insulin sensitivity and modify the susceptibility to increase weight. For a long time, BAT was only considered an energy-producing organ in rodents and newborns, undergoing involution with age. However, BAT has also been identified in human adults near the aorta and within the supraclavicular region of the neck. Nevertheless, the origin of BAT is still under debate [26, 29].

Recently, a type of AT showing intermediary characteristics between that of white and brown adipocytes, which has mixed structural features of both, was identified as beige AT [29]. This type of AT was reported as a set of adipocytes in WAT that might acquire a thermogenic phenotype with higher UCP1 expression, similar to brown adipocytes after enough stimulus [29, 30].

There are two major mechanisms described related to beige cells arising: d*e novo differentiation* which occurs from a progenitor resident cell and *transdifferentiation* which consist of differentiation of a mature white adipocyte through a molecular mechanism. The first theory is based on that beige adipocytes come from progenitor cells differentiation induced by adipogenic stimulation such as cold exposure, adrenergic signaling, exercise, natriuretic peptides, thyroid hormones, diets, and food components [31, 32]. Currently, several specific cell markers were identified in various types of progenitor cells such as in smooth muscle-like cells (Myh11^+^), preadipocytes (Pdgfrb^+^, SMA^+^), adipocytes progenitor cells (Sca-1^+^ Pdgfra^+^ CD81^+^) [33–35]. These adipogenic stimulation actives transcriptional machinery of browning that is characterized by the expression of Ucp1, Prdm16, Zfp516, and Pgc1a genes that will promote a beige differentiation [36].

On the other hand, the transdifferentiation hypothesis proposes beige cells arise from mature white adipocytes, in a reversible process, after adipogenic stimulus without the participation of a progenitor-like state of cells [37]. The underlying molecular mechanisms for transdifferentiation are under intensive research, but some studies already show that this plasticity process occurs mainly in scWAT depots [29]. Known as browning, this process has gained increasing attention in the research area as an alternative method of energy stimulation. UCP1 expression can be stimulated when white adipocytes are exposed to stimuli, previously referred as to adipogenic stimulus, [20, 27, 29], driven by a set of molecules known as browning markers.

### The uncoupling protein 1 (UCP-1)

The non-shivering thermogenesis is a phenomenon that occurs in brown and beige ATs due mostly to the action of UCP1 [38]. UCPs are transmembrane proteins that belong to the mitochondrial anion carrier family (MACF), i.e., mediate specific metabolite exchanges between the cell cytoplasm and the mitochondrial matrix and thus enable the activation of essential biochemical pathways [39, 40]. The UCPs exhibit 5 isoforms, ranging from UCP1 to UCP5 are present in several tissues [41, 42]. UCP1 is the main isoform associated with thermogenesis, it is widely and selectively expressed in the inner mitochondrial membrane of the adipocyte, representing about 10% of the total mitochondrial protein in human epicardial AT [43–46].

UCP1 protein is described as participating in thermogenesis by interfering in proton leakage within the chemiosmotic gradient during the mitochondrial oxidative phosphorylation by the translocating fatty acids (FAs). This gradient is obtained from the oxidation of substrates and provides the required force to induce the respiratory machinery to produce ATP. Once UCP1 promotes proton leakage, the energy obtained cannot be stored in the form of ATP and is alternatively dissipated as heat [47, 48]. Thus, it is evident that direct regulation of UCP1 protein activity is one of the means of regulating thermogenesis, and that occurs in opposite ways by cytosolic purine nucleotides and long-chain fatty acids (LCFA), promoting inhibition or activation of UCP1, respectively [49].

The other form of regulating UCP1 is at the transcriptional level. UCP1 gene is transcribed only in brown and beige adipocytes, associates with the differentiation state of these cells, and is quantitatively regulated in response to many physiological signals [9]. These characteristics are consequences of the transcriptional control mediated by trans-acting factors on regulatory regions found in the 5’ non-coding region of the UCP1 gene. The proximal regulatory region, which is found immediately upstream of the transcription start site, contains cAMP response element-binding protein (CREB) [50, 51] and CCAAT-enhancer-binding protein (C/EBP) [52] binding sites. Also in the proximity of the site of transcription start, activating transcription factor-2 (ATF2)-binding site interacts with transcriptional coregulators, such as PGC-1α, impacting UCP1 gene transcription [9]. In opposition to these proximal regulatory sites, a strong enhancer region is placed more than 2 kb upstream of the transcription initiation site [50, 53] and contains a cluster of response elements for nuclear hormone receptors [7, 54].

UCP1 gene activation and repression depend on which trans-acting factors bind to the regulatory region. For example, CREB binding sites mediate a positive transcriptional response to cAMP [50] and a negative response to AP2 (c-Jun/c- Fos) complexes [51]. Another example is the PPARγ binding site found in the distal enhancer region, which associates with gene activation after binding to its main ligand but represses UCP1 transcription when interacting with liver X receptor (LXR) and its corepressor receptor-interacting protein 140 (RIP140) [55]. RIP140 inhibits UCP1 gene transcription by enabling the assembly of DNA and histone methyltransferases on the UCP1 gene, altering the methylation status of CpG islands in the promoter region and histones, impacting gene expression through transcription machinery accessibility [56].

Although some epigenetic modifications are associated with repressed UCP1 gene expression, as in H3K9 demethylation marks, chromatin modifications indicative of activation of this gene also occur, such as in the case of H3K4 trimethylated marks, which are enriched in BAT [57]. Also participating in fine-tuning of gene expression, microRNAs (miRs) are characterized to be a group of short non-coding RNAs (ncRNAs) generated by the sequential processing of longer ribonucleic acid molecules [58]. While miR-328 [59] and miR-455 [60] are described to be activators of UCP1 gene expression, miR-27 [61, 62], and miR-133 [63] display UCP1 gene transcription inhibitory activity**.**

The roles of WAT and BAT in metabolic syndrome is well characterized, but the physiological and biochemical modulations of BAT remain unclear [64–66]. Several studies showed that UCP1-dependent BAT activity was mostly found to be beneficial in decreasing inflammation, and improving cardiometabolic homeostasis [67–69]. However, this tissue has a lower activity in obese in comparison to healthy individuals [70].

It is well established that the deficiency of the UCP1 gene is not enough to protect against diet-induced obesity (DIO), but can modulate important physiological and metabolic parameters in mice [64, 65]. The food intake-induced browning is inhibited in the absence of UCP1, demonstrating the intimate relationship between this differentiation process and UCP1 [71, 72]. More than that, UCP1 -/- female mice fed with a western diet displayed increased whitening of BAT, as well as metabolic disruption indicated by glucose intolerance, upregulation of genes related to inflammation, liver steatosis, immune cell infiltration, and endoplasmic reticulum (ER)/oxidative stress [73]. Accordingly, even during standard diet under cold exposure, UCP1−/−, male mice showed BAT immune cell infiltration and ER stress profile [74]. The lack of UCP1 promotes de novo lipogenesis and hyperplasia of inguinal WAT, leading to an increase in FA trafficking to the liver [75].

In contrast, the upregulation of UCP1 or even only its activation can perform a paradoxical role in hypermetabolic scenarios and associate with a worse prognosis [76, 77]. It is proven that diet-induced whitening is related to the upregulation of this gene. A greater expression of browning markers (e.g., UCP1, PGC-1α, TBX1) was found in obese human patients mainly in vWAT [78] Besides that, mice affected by cancer-associated cachexia tend to show increased thermogenesis gene expression and BAT activation [79].

The browning process is spontaneously induced by tumor-secreted factors and IL-6 during cachexia development, which can lead to full depletion of AT [80, 81]. Interestingly, the elicitation of browning after burn injury is associated with the hypermetabolic response, as well as an increase in lipolysis and free fatty acid efflux that can outcome in liver steatosis [82, 83]. In addition to the therapeutic impact of the browning process in obesity and metabolic diseases, recent discoveries regarding the impact of UCP1-dependent BAT activity in hypermetabolism conditions should be further investigated in the context of UCP1 to appropriately regulate browning for application in different situations.

### Beta 3 adrenergic receptor activation

Increasing energy expenditure through activation of BAT shows potential for treating metabolic diseases, and that is the reason this approach has been deeply investigated [84]. The white/brown plasticity of ATs and tissue thermogenesis appear to be activated by a β-3 adrenergic receptor (β-3AR) system [4, 30, 85]. β3-ARs are expressed predominantly on white and brown adipocytes [86]. Murine WAT expresses β3-AR transcripts in a greater proportion compared to other β-ARs, similar to BAT [87]. Although β3-AR mRNA levels are lower in humans than in rodent AT, its roles seem to be fundamental in the regulation of energy balance and glucose homeostasis [88].

Browning of WAT occurs mainly by noradrenaline and adrenaline stimulation, which influence lipolysis after binding to different adrenoceptor subtypes on the cell-surface membrane of fat cells. The interaction with β3-AR initiates a cascade of signal transduction that ends with the overexpression of thermogenic proteins, such as UCP-1 [88, 89]. The adaptive thermogenic response is initiated by the central (CNS) and sympathetic (SNS) nervous systems with the release of norepinephrine (NE) and stimulation of β3-AR, through the G protein-coupled receptor Gs, which in turn activates the adenylyl cyclase (AC), stimulating the production of cyclic adenosine monophosphate (cAMP), and activating the protein kinase A (PKA) pathway. Then, these signals from the cAMP pathway, finally, upregulate UCP-1 and lipolysis [88–92].

A distinguishing feature of the β3-AR, already seen in past studies, is that it appears to be relatively resistant to desensitization and down-regulation, leading to the hypothesis that one of its functions might be to maintain signaling during periods of sustained sympathetic stimulation, as in diet-associated β3-AR activation or cold exposure [87]. Cold temperature exposure elicits a coordinated physiological response aimed at maintaining their body temperature. This response activates the mentioned cascade and generates heat in beige adipocytes within scWAT and BAT [85]. Thus, it was seen that mice with a combined target disruption of the three β1, β2, and β3 adrenergic receptors (TKO mice) have increased susceptibility to cold-induced hypothermia as well as diet-induced obesity [91]. Thereby, mice β3-AR activation started to be studied, effectively mimicking cold exposure effects [84, 91].

Initial studies demonstrated that WAT UCP1 mRNA and protein levels are strongly decreased in β3-AR knockout (KO) mice [30, 93]. In addition, β3-AR agonists are well-known for inducing ectopic UCP1 expression in WAT coupled with a significant mitochondrial enhancement in rodents, and for augmenting glucose homeostatic activity of their BAT [84, 94]. On the other hand, in humans, early efforts to increase browning activation with the use of β3-adrenoreceptor agonists have failed in clinical trials because of their β1- and β2-AR-mediated cardiovascular effects [13, 84, 94]. However, a recent study showed that mirabegron, a selective β3-agonist previously developed for the treatment of overactive bladder, was shown to increase BAT activity as compared to placebo. This study used an oral dose of 200 mg in healthy male subjects, and despite not having severe cardiovascular side effects, they have been shown to increase heart rate and systolic blood pressure [84]. That is the reason long-term studies are warranted to investigate the effectiveness and cardiovascular safety of this type of treatment to induce weight loss and metabolic health improvements.

Genetic factors must be considered in influencing adipocyte lipolysis regulation. Genetic variance in β3-AR and its specific G-coupling protein has functional effects on lipolysis. Polymorphism in the G-β3 gene, for example, influences catecholamine-induced lipolysis in human fat cells by altering the coupling of β3-AR to G-proteins [88]. This proves once again the importance of the β3-AR presence for the thermogenic process.

### Temperature-induced browning

It is well established that temperature can modulate biochemical, inflammatory, and immunological processes systemically, displaying relevant physiological impact [95, 96]. Despite this, the influence of warmer temperatures is better described compared to cold conditions due to their immediate danger. Fever, triggered by infectious and inflammatory processes, was associated with a worse prognosis in the past centuries, demanding greater medical attention for a long time [97]. However, currently, it is recognized that both hyper and hypothermia, in properly regulated circumstances, are beneficial response mechanisms to infection in mild and severe profiles, respectively [98].

Hypothermia is also associated with an advantageous mechanism against severe systemic inflammation. In experimental studies, the infectious or aseptic systemic inflammation process is elicited by the intravenous administration of bacterial lipopolysaccharide (LPS) in mice [99–101]. The variances of body temperature are modulated by the environmental temperature and concentration of LPS introduced [102]. Animals housed in hyperthermal conditions or exposed to lower LPS concentrations displayed polyphasic fever. In thermoneutral conditions, the fever was also usually elicited to induce the immunological response. However, if the mice were housed in cooler acclimation or administered with higher LPS concentrations, the effect elicited was hypothermia, which associates with arterial hypotension aimed to avoid infection spread, followed by polyphasic fever.

The ideal body temperature is obtained by the modulation of blood vessel tension degree, and heat production by thermogenesis. Cutaneous vasoconstriction and thermogenesis processes occur to increase body temperature and avoid heat loss. Conversely, the opposite effect, skin vasodilatation and thermogenesis inhibition, is stimulated to induce hypothermia [102–105].

Temperature is a paradoxical agent with important roles not only in biological events but also in the development of several diseases [106–110]. Hypothermia, specifically, displays a typical profile, in which the energy is preserved. The decrease in body temperature also favors the development of an anti-inflammatory profile and immunosuppression, which can act as a double-edged sword depending on the condition [108, 111–113]. In the tumoral context, hypothermia provides an immunosuppressive, hence, pro-tumoral microenvironmental due to impaired CD8+ T cell function, an increase of the regulatory and Th2 cells, and higher levels of the cytokines IL-4 and IL-10, which increase cancer progression and metastasis [114–116].On the other hand, hyperthermia is recognized to elicit a more robust immune response against infection, injury, and cancer [117]. Several studies reported an increase in IFN-γ and IL-2 secretion from peripheric T cells, enhancement of cytotoxicity, DC maturation, and increase of tumor-specific CD8+ T cells [118–121].

In inflammatory conditions, such as neurological damage, atherosclerosis, systemic inflammation, and hypothermia (cryotherapy) can be beneficial [122–126]. Cryotherapy benefits are illustrated by an experimental approach that submitted healthy and physical activity practitioners men to intense exercises aimed to induce muscle injury [127]. It was observed that cryotherapy mediated the increase of IL-10, reduction of pro-inflammatory cytokine IL-1, reduction of muscle damage and blood cholesterol, decrease oxidative stress and improve the lipid profile not only in healthy patients but also in patients with active-phase ankylosing spondylitis [124, 128, 129]. Cryotherapy also shows to be neuroprotective capacity, alleviating sequelae from ischemic or hemorrhage stroke, cardiac arrest, intracranial pressure elevation, and traumatic brain injury [130].

In the same line, recent research evaluated the impact of spontaneous body hyperthermia after brain injury. Metabolic modulations were observed as the diminishment of both cerebral and arterial glucose levels and increase of lactate-pyruvate ratio. However, these changes were not associated with a worse prognostic [131]. Additionally, induced hyperthermia in healthy men promoted an increase in cerebral metabolic rate of oxygen (CMRO_2_), also increase IL-6 and myeloperoxidase (MPO) systemically, but did not promote the same inflammatory and oxidative phenomenon in the brain [132]. In peripheral organs such as the liver, hyperthermia is associated with an increase in oxidative metabolism, vasodilation, and an increase of heat shock proteins (HSP) expression. HSP displays an important role in metabolism such as modulation of both glucose and lipid metabolism in the liver and improving the mitochondrial skeletal muscle functionality [133].

In addition, temperature modulates directly the shivering and non-shivering thermogenesis processes. The occurrence of these events maintains proper body temperature under adverse thermal acclimation. Once shivering thermogenesis is decreased in cold acclimation (around 4 °C), non-shivering thermogenesis is the major way to produce heat in this context [2, 4]. The detection of the thermal changes begins with the capture of sensory stimuli by cutaneous thermoreceptors, which promote the sensitization of afferent nerves. The stimuli are directed to the CNS, which then induces thermoregulatory responses, including vasoconstriction and catecholamines secretion. These catecholamines, mainly NE, increase BAT activation, hence heat production through a UCP1-dependent manner [134, 135].

BAT is a highly innervated and vascularized organ that displays considerable amounts of β3-ARs, which is also expressed in WAT, though at a lower level. NE binding to β3-ARs promotes systemic adrenergic activation, which induces a signal cascade culminating in the accumulation of adipokines, such as Zinc-α2-glycoprotein (ZAG), increase in thermogenesis-related gene expression, as UCP1, thus enabling mobilization and oxidation of free fatty acids (FFAs) in both tissues, increasing BAT activity and promoting browning in WAT. [84, 136, 137].

It is known that the results observed in humans do not always represent the same effects previously described in mice or even contradictory results can be obtained under similar conditions for the same species [138]. Unfortunately, this premise can also be applied to cold-induced browning. Leitner and colleagues showed that in human fewer than half of the BAT deposits is stimulated by cold exposure, hence, the thermogenic function was lower than expected [44].

Brychta and others demonstrated that the profile of men with obesity was associated with a reduced tolerance limit to chill temperatures, suggesting that thermogenesis was diminished in these individuals, as well as energy expenditure [139]. Blauw et al. [140] demonstrated a surprising association between the impairment of glycemic homeostasis, diabetes, and obesity in humans housed in the United States of America (USA) at warmer temperatures and associated these results with a decrease in BAT activity. Taking the assessment on a global scale, Kanazawa evaluated the parallel between higher temperatures, weight gain, and obesity. The data analyzed allowed to predict that global warming could be responsible for an increase of more than 10% in the obesity rates in 120 years, counting from 1961 [141].

Notably, the use of cold as a browning inducer has been carefully applied not only because of the side effects that can be displayed at the whole-body level but also due to the contradictory effect observed in humans. If on one hand, the anti-inflammatory and immunosuppressive role mediated by cold and cold-induced browning is beneficial in healthy individuals, for ill people these same effects may become harmful. In contrast, intriguing research has brought a new perspective on temperature-based browning. Li and colleagues discovered that a hydrogel-based photothermal therapy leads to a successful increase of beige activation in both mice and humans. The therapy consists of increasing the local temperature, around 41˚C, without evident stress on skin or adjacent tissues [142]. This promising study succeeds previous findings that pointed to the occurrence of WAT browning after burn injury [143, 144]. The characterization of possible inducers of browning is a strongly growing field since the applicability of these inducers as therapy in humans has proven to be a major clinical hurdle. However, even under promising advances is clear that further investigation regarding the mechanisms triggered by this stimulus pathway should be conducted.

### Exercise-induced browning

Physical exercising is already associated with improvements in several processes related to the cardiovascular system, skeletal muscle, and ATs [193]. Following this, several studies show that physical activity provides better quality of life [194] and helps in the treatment of several metabolic diseases and obesity [195] through increasing AT lipolysis, vascularization, blood flow, and promoting the secretion of hormones and adipokines [196]. Among the main adipokines, FGF21 (Fibroblast growth factor 21) and leptin stand out, which act in an autocrine/paracrine manner, regulating WAT browning process [197]. After physical activities, the adipokine leptin stimulates activity in the sympathetic nerve and together with insulin act synergistically in different neuronal subsets of proopiomelanocortin (POMC) inducing browning of WAT through decreased hypothalamic inflammation caused by exercise [198]. During exercise, the increase in glucagon, which already has thermogenic potential [199], and the decrease in insulin in the liver lead to FGF21 secretion [200].

The exercise induces pleiotropic effects in the liver, AT, immune system, and skeletal muscle by enabling myokine secretion upon contraction [201]. After activities, muscle cells increase the expression of PGC-1α, inducing BAT thermogenesis and mitochondrial biogenesis. Among the myokines involved in the browning process, interleukin-6 (IL-6) is a modulatory cytokine secreted by several tissues, including skeletal muscle and AT. A study showed that mouse AT when treated with IL-6 for 6 h induces the expression of PGC-1α and mitochondrial enzymes [202]. In addition, analyses showed that IL-6 is involved in the increase of UCP1 mRNA in inguinal WAT (igWAT) stimulated by physical activity [203]. Another relevant myokine is Irisin; Once exercising increases the expression of PGC-1α, it induces increased levels of the fibronectin domain-containing protein 5 (FNDC5) protein, which, after being cleaved, is released as the hormone irisin [204]. Irisin was shown to stimulate UCP1 expression and thermogenic differentiation of white fat precursor cells in vitro and in vivo [145].

The myokine myostatin (Mstn), a growth factor that limits muscle growth and development, is negatively involved in the WAT browning process, as Mstn-deficient mice showed high expression of genes associated with FA oxidation, mitochondrial biogenesis, lipid transport together with the positive regulation of PGC-1α and UCP1, this mechanism occurs through the phosphorylation of AMPK, necessary for the activation of PGC1α and FNDC5 [199]. Metrnl, the gene encoding for Meteorin-like protein, is a myokine known to be induced by resistance exercise dependently on PGC-1α4. Metrnl regulates genes involved in thermogenesis, as it is capable of promoting the activation of M2 macrophages by inciting the expression of IL-4 and thus triggering the production of catecholamines [206], responsible for favoring thermogenesis in AT [207]. Beta-Aminoisobutyric acid is another myokine that has increased levels during exercise and can induce the brown adipocyte phenotype in human-induced pluripotent stem cells during differentiation to mature white adipocytes [146].

Intense physical activity causes increased heart rate and stretching of cardiomyocytes, which cause the secretion of atrial natriuretic peptide (ANP) and brain natriuretic peptide (BNP), molecules that stimulate lipolysis, UCP1 expression, and mitochondrial biogenesis [208]. It also induces an increase in lactate, which binds to receptor GRP81 on adipocytes, leads to an increase in P38 phosphorylation, and thus mediates the browning of WAT by activating the PGC-1a, PPAR, γ, and Ucp1 genes [147]. During lipolysis, FAs are not only used as an energy source but also undergo the re-esterification process where they are converted into triglycerides in AT. This re-esterification consumes ATP generating AMP. AMP in turn can activate AMPK, which then induces greater expression of PGC-1α and mitochondrial biogenesis [210].Another the important effect induced by exercise that plays an important role in the browning of WAT is oxidative stress in skeletal muscle, whish it responsible for the increase in H_2_O_2_ through the reduction of glutathione levels, a molecule capable of supplying electrons to glutathione peroxidase, thus increasing H_2_O_2_ levels. And also by increasing the activity of superoxide dismutase 2 (SOD2), which reduces ROS to H_2_O_2_. When H_2_O_2_ enters the circulation, it is directed to WAT and subsequently induces the expression of thermogenic genes [148]. Exercise also increases the level of succinate, resulting in augmented levels of mitochondrial reactive oxygen species, which in turn promotes the sulphenylation of Cys253 to increase UCP1 activity [149].

Although the studies conducted in mice seem promising, the effect of exercise on WAT browning in humans has proven to be controversial. A survey conducted with sedentary subjects participating in a 12-week bicycle-training program showed scWAT increased expression of UCP1, carnitine palmitoyltransferase 1B (CPT1B), TBX1 [15]. However, other studies have not achieved similar effects. Tsiloulis and colleagues collected scWAT of obese men after 6 weeks of physical training and the mRNA levels of UCP1, CD137, CITED, TBX1, LHX8, and TCF21 were not altered [211]. Many factors may be involved in this diversity of results since the duration, frequency, and degree of intensity are associated with these effects. Thus, more human studies need to be conducted as many questions still need to be clarified.

### Fibroblast growth factor 21

The fibroblast growth factor family (FGF) performs a range of cellular metabolic and physiological responses to maintain overall homeostasis. FGF 21 was first identified in mice and humans in 2,000 by Nishimura and colleagues through cDNA identification in different organs [150]. While the gene in mice is located in chromosome 7 and encodes a preprotein of 210 amino acids (aa), in humans it is found in chromosome 19 and encodes a preprotein of 209 aa. Proteins belonging to the FGF family exert a wide range of functions, from promoting cell proliferation and differentiation to systemic effects [151, 152], acting as autocrine/paracrine/endocrine factors [153]. Most FGF family members have a high affinity to heparin sulfate, except for the endocrine FGF (FGF) subgroup, which consists of FGF 19 (FGF 15 in rodents), FGF 21 e FGF 23 in humans [151]. FGF molecules lack an extracellular heparin-binding domain and thus can enter the blood system [154].

FGF 21 binds to a fibroblast growth factor tyrosine kinase receptor (FGFR), which can be found in seven isoforms: 1b, 1c, 2b, 2c, 3b, 3c, and 4. The FGF 21 requires its dimerization with a klotho protein, called beta-klotho (KLB). Thus, the FGFR-KLB receptors lead to the intracellular cascade that goes through the phosphorylation of FGFR substrate 2α (FRS2α) and the activation of Ras-MAPKs and PI3K-Akt kinases [154–156]. Once FGF21 signaling requires KLB to activate FGFRs, the co-expression of these two receptors determines the sensitivity of a tissue or organ to FGF21 [157]. FGF 21 is defined as a stress-responsive hormone [152], which effect is subtle in physiological conditions but significantly exacerbated under nutritional, metabolic, oxidative, hormonal, or environmental challenges. Consequently, starvation and overfeeding, ketogenic and high-carbohydrate diets, physical exercises, protein restriction [158], type 2 diabetes (T2D), obesity [153], and nonalcoholic fatty liver disease (NAFLD) [159] can induce the expression or/and signaling of FGF 21 [158].

FGF 21 is synthesized mainly in the liver and thymus but is also detected in skeletal muscle, pancreas, intestine, heart, β cells, and WAT and BAT [160]. As an important metabolic regulator, acting mostly in glucose and lipid homeostasis, FGF 21 triggers lipolysis and FFAs released in circulation from WAT during prolonged fasting or starvation [29, 160]. PPAR-α is activated in the presence of FFA and improves FFA oxidation and ketone bodies formation for acting as energy sources during prolonged fasting. The interaction between FFA/PPAR-α/retinoid X receptor (RXR) has a PPAR-α response element, which activates FGF 21 promoter [161, 162]. Thus, when PPAR-α activity increases, the production of FGF 21 in the liver also augments, leading to energy production, increased ketogenesis, gluconeogenesis, appetite, and systemic glucose uptake as adaptive responses to starvation [163]. The activity of FGF 21 is not limited to starvation conditions, but it is also increased in adaptation to high-fat (HF) intake [164].

Human studies inform that FGF21 production is stimulated in situations of decreased thermogenesis, reduction in adiponectin levels, and tissue breakdown markers, such as transaminases elevation mare than changes in levels of FFAs [165]. Another means of increasing FGF21 levels, through PPAR-α activity, is through intense physical activity, growth hormone therapy, lactation, and milk ingestion in neonates [163, 166]. Macronutrients such as proteins also regulate FGF 21 production through amino acid restriction [167]. This process starts when the general control non-derepressible 2 (GCN2)-eukaryotic initiation factor 2 (eIF2) α pathway is activated inducing the binding of activating transcription factor 4 (ATF4) to PGC-1 α [168, 169].

After being secreted, its most important target is WAT, where FGF21 improves insulin sensitivity [153, 160] and increments GLUT1 expression and consequently glucose uptake, as shown by in vitro 3T3-L1 adipocyte analyses [153, 170]. The response element-binding protein (ChREBP) is sensitive to carbohydrates in the liver and ChREBP interaction with PPAR-γ in adipocytes modulates the expression of FGF21. In other words, the upregulation of ChREBP may induce the expression of this FGF [160]. Another example of FGF 21 influence on carbohydrate metabolism is through the suppression of hepatic pyruvate dehydrogenase (PD) complex through PD kinase 4 activity [171]. Additional transcription factors, such as retinoic acid (RA) receptor β (RARβ), TRβ, cyclic AMP response element-binding protein H (CREBH), RA receptor-related orphan receptor α (RORα), respond to determinants in the liver and regulates FGF 21 production [151].

WAT is not only a target of FGF21, but it is the major mediator of its effects. The processes of glucose- and insulin-sensitive responses depend on adiponectin production and secretion by this tissue [172]. Adiponectin also reduces the levels of sphingolipid ceramides in obese animals, which have been associated with lipotoxicity [173]. The action of FGF21 in WAT includes paracrine and autocrine actions and is mediated through the induction of PGC-1α protein in cold and through the enhanced levels of the thermogenic protein UCP1, which is a key protein for heat production [174]. BAT requires the FGFR1/KLB complex to respond to FGF21, which induces glucose uptake and thermogenesis through the induction of UCP1, in response to its autocrine and paracrine production. FGF 21 impact derives from increased PGC-1α levels and, consequently, expression of UCP1 [174]. In conclusion, FGF 21 is involved in glucose uptake, lipogenesis, and lipolysis, depending on the metabolic state of the adipocytes. This dual phenomenon may depend on nutritional condition, FGF21 concentrations reached between pharmacological administration and physiological secretion [175].

### Thyroid-hormone-induced browning

Thyroid Hormone (TH) is essential for metabolism in mammals and associates with many processes, including organism development, metabolic regulation, neural differentiation, and growth [176]. Many genes are regulated after its conversion from the prohormone thyroxine (T4) to the activated form triiodothyronine (T3) [177] by 5′-deiodinase type 2 (D2), enzyme known to be expressed in the hypothalamus, WAT, BAT, and skeletal muscle, and to be required for adaptive thermogenesis [178]. TH is produced in the follicles of the thyroid gland and is synthesized through iodination of tyrosine residues in the glycoprotein thyroglobulin [179, 180]. The main means of regulator its production is through thyroid-stimulating hormone (TSH), which binds to the TSH receptor (TSH-R) expressed in the thyroid follicular cell basolateral membrane and is released by the anterior pituitary in response to a circulating TH [181].

The biological response of TH is complex and highly regulated. It is mediated by thyroid hormone nuclear receptors (TRs). The TR genes produce two main types of receptors, α and β, and their isoforms α1, α2, α3, β1, β2, and β3, but only α1, β1, β2, and β3 are T3-binding receptors, which are differentially expressed in tissues and have distinct roles in TH signaling [178, 182]. TH enters the cell through membrane proteins monocarboxylate transporter 8 (MCT8) and solute carrier organic anion transporter family member 1C1 9 (OATP1C1), then interacts with TR in the nucleus, which binds to the genomic thyroid-hormone responsive elements (TREs) and other nuclear proteins, including corepressors, coactivators, and cointegrators, leading to chromatin remodeling and the regulation of the UCP1 gene transcription [183, 184].

This hormone is correlated with weight and energy expenditure. Thus, hypothyroidism, characterized by diminished TH levels, leads to hypometabolism, a condition associated with reduced resting energy expenditure, weight gain, high cholesterol levels, reduced lipolysis, and gluconeogenesis. On the other hand, hyperthyroidism, and elevated TH levels, induce a hypermetabolic state, characterized by increased resting energy expenditure, lower cholesterol levels, increased lipolysis and gluconeogenesis, and weight loss. Consequently, TH controls energy balance by regulating energy storage and expenditure regulating key metabolic pathways [178].

TH regulates basal metabolic rate (BMR) through ATP production, used for metabolic processes, and by generating and maintaining ion gradient [185–187]. BMR is induced by the stimulation of two main gradients, the Na+/K+ gradient across the cell membrane and the Ca^2+^ gradient between the cytoplasm and sarcoplasmic reticulum and produces heat during ATP hydrolysis [188]. TH maintains the BMR levels through the uncoupling oxidative phosphorylation in the mitochondria. When ATP production is compromised in skeletal muscle, TH increases the leak of protons through the mitochondrial inner membrane, stimulating more oxidation to maintain ATP synthesis [189].

TH regulates metabolism primarily through actions in the brain, WAT, BAT, skeletal muscle, liver, and pancreas [178]. This action, as already said, is through TH receptors (TR) isoforms, WAT has the adrenergic signaling increased by TRα [190], otherwise BAT expresses TR α and β, as it needs TRα for adrenergic stimulation and TRβ for stimulating of UCP1, both for thermogenesis [176]. In humans, T3 administration induced UCP1 expression, dependent on the presence of TRβ, which induces “browning” [191]. TH regulates several aspects of lipid metabolism and human BAT from lipogenesis to lipoprotein signaling [192]. Rats administrated with T3 showed how the central nervous system is important to the activation of BAT by TH through inhibition of hypothalamic AMP-activated protein kinase (AMPK). Stimulation of sympathetic nervous system (SNS) activity leads to thermogenic gene expression in BAT [193].

As discussed previously, β-AR is stimulated by NE in response to SNS [1]. The expression of UCP1, required for BAT thermogenesis, is regulated by NE and T3 synergistically, once the induction in separate is twofold, while combined is 20 -fold [194]. Another way that UCP1 expression and thermogenesis are induced is through bile acid stimulation. G protein-coupled membrane bile acid receptor (TGR5) is stimulated in BAT and results in D2 stimulation and local T3 production [178].

In conclusion, several mechanisms have been proposed for the TH influence in the browning process, including cold exposure, adrenergic activation [167], and bile acid signal [178]. Thus, the stimulation of BAT activation and WAT browning increase the energy expenditure, loss of weight [195], D2 activation, UCP1 level increase, and consequent thermogenesis [192].

### Circadian rhythm and browning

As previously discussed here, several exogenous factors are able to elicit browning of WAT and BAT activation. However, endogenous factors also play an important role in regulating the phenotype and physiology of these tissues. One of the most important endogenous factors that are related to the regulation of AT is the circadian rhythm, which is a refined system that acts as a master biological clock synchronizing daily and seasonal variations with the behavioral, cellular and tissue-autonomous clock, as well as several biological processes that include sleep–wake cycle, hormone secretion, lipid and glucose homeostasis, energy balance and body temperature [196]. The circadian rhythm is controlled by melatonin synthesis, which can occur both in the CNS, more specifically in the pineal gland being regulated by light/dark stimulus via the suprachiasmatic nucleus of the hypothalamus (SCN), and in peripheral tissues where its regulation remains unclear [197].

Disruption of circadian rhythm caused by aging, shift-work, irregular sleep, insomnia, or long exposure to light during the night is associated with sleep and metabolic disorders such as cardiovascular diseases, diabetes type 2 and obesity. Regarding metabolic diseases, AT plays a central role in metabolic and whole-body energy homeostasis, once its secretes several adipokines that regulate diverse processes in CNS and peripheral tissues. Leptin, a hormone mainly produced by adipocytes, is released into the circulation where it crosses the blood–brain barrier (BBB), through a saturable system, and interact with its receptor in the hypothalamus LepRb [198, 199]. Hsuchou and colleagues demonstrated that leptin signaling disruption through a pan-leptin receptor knockout (POKO) in mice was able to dysregulate feeding behavior, metabolic and circadian rhythm profile and thus promote an accentuating of obesity [200]. Beyond control of feeding and metabolic processes, leptin also displays a role in energy balance through the increase of AT thermogenesis in BAT by sympathetic activation [201, 202].

Recent studies have proposed that diurnal rhythm promotes differential modulation in activity, thermogenesis and fat oxidation in BAT. It was observed that plasmatic lipid metabolism was improved during daytime with a higher expression of lipoprotein lipase, FA uptake, and modulates lipid plasmatic concentration in BAT [203]. In the same line, Matsushita and colleagues, assessed forty-four healthy men who received diet-induced thermogenesis (DIT) under room temperature (27 °C) and cold (19 °C) in the morning and in the evening by using ^18^F-fluoro-2-deoxy-D-glucose positron emission tomography. It was observed that thermogenic parameters presented better performance during the morning [204].

Moreover, several studies have established that melatonin directly impacts BAT morphology and function, also, in a mechanism dependent on adrenergic activation mediated by NE release. Melatonin is related to an increase of BAT volume, and thermogenic capacity, associated with the increase of UCP1 mRNA expression and mitochondrial mass and functionality, as well as seric lipid concentration. These profiles are significatively impaired under melatonin deficiency but reverse with oral melatonin replacement [205–207]. Growing evidence confirms the intimate relationship between circadian rhythm and AT, with emphasis on metabolic homeostasis and modulation of BAT activity. The characterization of how this process happens emerges as a strong diagnostic tool as well as a therapeutic approach concerning sleep disorders and metabolic diseases.

### Food-intake and browning

Several studies suggest that food items can affect AT function. Among them curcumin, present in saffron, proved to be involved in the browning process, as mice treated with 50 or 100 mg/kg/day of this compound increased inguinal WAT expression of several browning-associated genes, such as Ucp1, Pgc1a, Prdm16, Dio2, PPARα, and CIDEA, and displayed mitochondrial biogenesis in this tissue. Curcumin stimulation was unable to induce the same effects in the epididymal WAT, though. This process was mediated by the NE-β3-AR pathway since the levels of NE and β3-AR were elevated in the inguinal WAT [208]. Although studies are scarce regarding the impact of thyme in the WAT browning process, it was observed that 20 µM of thymol, a substance present in the essential oils of thyme, in the complete medium when placed in contact with 3T3-LI preadipocytes for 6–8 days was able to induce an increased gene and protein expression of the PGC-1α, PPARγ, and UCP1. Such increases were related to the activation of β3-AR, AMPK, PKA, and Mitogen-activated protein kinase (p38 MAPK) being accompanied by an increase in mitochondrial biogenesis [209].

Cinnamon oil contains trans-cinnamic acid, which exposure to 3T3-L1 white adipocytes at 100 µM high gene expression of Lhx8, Ppargc1, Prdm16, Ucp1, and Zic1 and markers of UCP1, PRDM16, and PGC-1α, indicating WAT browning [210]. Quercetin, a flavonoid present in the onion, also proved to be efficient in the browning process since mice fed for 8 days with 0.5% onion peel extract (OPE) during a high-fat diet (HFD) exhibited increased expression levels OF UCP-1, PRMD16, AND PGC1-α in retroperitoneal white adipose tissue (rWAT) [211]. Just as the combination of quercetin and resveratrol also induces the WAT browning phenotype [212]. The resveratrol, present in the bark of grapes and other plants, also increases the expression of UCP-1, PRDM16, and PPARγ, suggesting that resveratrol induces the formation of beige adipocytes through the phosphorylation of AMPK, once treatment coupled with inhibition or the deletion of AMPK did not produce the same effects [208]. The same was observed in the substances found in the mushroom and honey, which induced increased expression of brown fat markers via AMPK and PGC-1α [213].

The peppers have capsaicin, an active compound responsible for the burning sensation that is also involved in the browning of WAT. One study used wild-type (WT) and TRPV1^−/−^ mice fed with HFD containing 0.01% capsaicin. The WT animals showed an increase in the expression of Ucp-1, Pgc-1α, Sirt-1, Prdm16, and exhibited browning of WAT via activation of the transient receptor potential vanilloid 1(TRPV1), which is related to the synthesis of catecholamine or sirtuin 1 (SIRT1)-mediated deacetylation of PPARγ, facilitating PPARγ-PRDM-16 interaction. The same did not happen in animals TRPV1^−/−^, demonstrating that capsaicin depends on the role of TRPV1 in the browning process [214]. Menthol, an organic compound extracted from *Mentha piperite* oil, was shown to activate Transient Receptor Potential Cation Channel Subfamily M Member 8 (TRPM8), and this matched the increase in the thermogenic Ucp1 gene and the expression of Pgc-1α through PKA phosphorylation induced by free intracellular Ca^2+^ in adipocytes treated with menthol for 8 h [215]. Other substances, such as carotenoids, are involved in the WAT browning process. Fucoxanthin, β-carotene, and citrus fruits are efficient in modulating the Ucp1 expression (216–218).

Another food component that is involved in the browning process of WAT is berberine, a molecule derived from the plants *Coptis chinensis* and *Hydrastis canadensis*. Obese male C57BLKS/J-Leprdb/Leprdb mice (db/db) were injected for 4 weeks with berberine (5 mg/kg/day). The group discovered berberine promotes BAT thermogenesis and WAT browning, since the igWAT, but not the epididymal, showed high levels of mRNA and UCP1 protein expression and increased mitochondrial biogenesis after injections. The brown adipocyte markers PGC-1α, CIDEA, Cox8b, and lsdp5 were also elevated and AMPK and PGC-1α are involved [219]. In another study, the polyphenols from tea extracts (0.5%) present in the high-fat diet for 8 weeks reduced the size of adipocytes and induced browning markers in WAT, and the size of lipid droplets and whitening markers were reduced in the BAT [220]. Another analysis with the extract induced 77.5 or 155 mg/kg/day for 8 days, in which there was an increase in UCP-1 and PPARγ [221].

Genistein, present in soy, indirectly induces browning since it is capable of increasing irisin levels, through PGC-1α / FNDC5, which increases Ucp1 and Tmem26 expression (222). In Magnolia Officinalis, two magnolol compounds (20 µM) and Honokiol (1–20 µM) when used to stimulate 3T3-L1 adipocytes increased protein levels of PGC-1α, PRDM16, and UCP-1 [223]. Honokiol also increased protein expression levels of CIDEA, COX8, FGF21, PGC-1α, and UCP1 [224]. The herb panax ginseng contains ginsenoside Rg1 (10 μM of ginsenoside Rb1), which is capable of considerably increasing the mRNA expression of UCP1, PGC-1α, and PRDM16 in mature 3T3-L1 adipocytes via PPARγ [225], as well as activating the AMP-activated protein kinase pathway [226].

The fish oil is rich in n-3 polyunsaturated fatty acids (PUFAs), components that are associated with the formation of beige adipocytes, among them is eicosapentaenoic acid (EPA). Mice fed different diets, including with EPA, for 8 weeks showed increased expression of β3-AR, PGC-1α, and UCP1 and exhibited high expression of PPAR [227], though this effect is controversial since another animal study investigating a diet containing pure EPA (3.6% as EPA ethyl ester) did not show the expression of beige adipocyte marker genes of inguinal and visceral WAT, but only in BAT [228]. Docosahexaenoic acid (DHA) (1.2%) together with EPA (2.4%) increased oxygen consumption and rectal temperature, as well as UCP1 and β3AR levels via the central nervous system. However, knockout mice for TRPV1 did not achieve the same effect, showing that such events were mediated by SNS, TRPV1, and catecholamines [229]. Conjugated linoleic acids (CLAs) also showed potential to induce browning process in the WAT [230].

Once the overwhelming impact of infectious diseases has been alleviated by the development of efficient therapeutics, life expectancy has been continuously increasing (World Health Organization, 2019). Age-associated diseases, including type 2 diabetes (T2D), cardiovascular diseases (CVDs), neurodegenerative pathologies, and obesity statistics are alarming and correlates with changes in the lifestyle of individuals throughout the world, including the diet, and impair the health spam rise. Western diets (WDs) are composed by food items enriched in processed sugar, white flour and salt and poor in fibers, vitamins and minerals [231]. At the same time, the diet may be the remedy against the burden caused by these chronic diseases. While overnutrition often correlates with inflammatory and metabolic detrimental effects at molecular level, undernutrition without starvation presents many benefits. Calorie restriction (CR) and intermittent fasting (IF) are promising interventions against the overweight and obesity numbers, climbing specially in Western countries [232].

CR, defined as reduced calorie consumption without malnutrition, is the best studied dietary intervention that increase health spam in experimental models. A plethora of human studies place CR as beneficial for expanding the health spam [232]. These studies proceeded Weindruch and Sohal positive correlations between CR and health spam [233] Click or tap here to enter text.. AT plasticity is one of the connections between CR and health benefits. Fabbiano and colleagues analyzed mice under CR and described that this regimen induces functional beige fat development in WAT, phenomenon that occur via enhanced type 2 immune response and SIRT1 expression in AT macrophages [234].

**Fig. 2:**
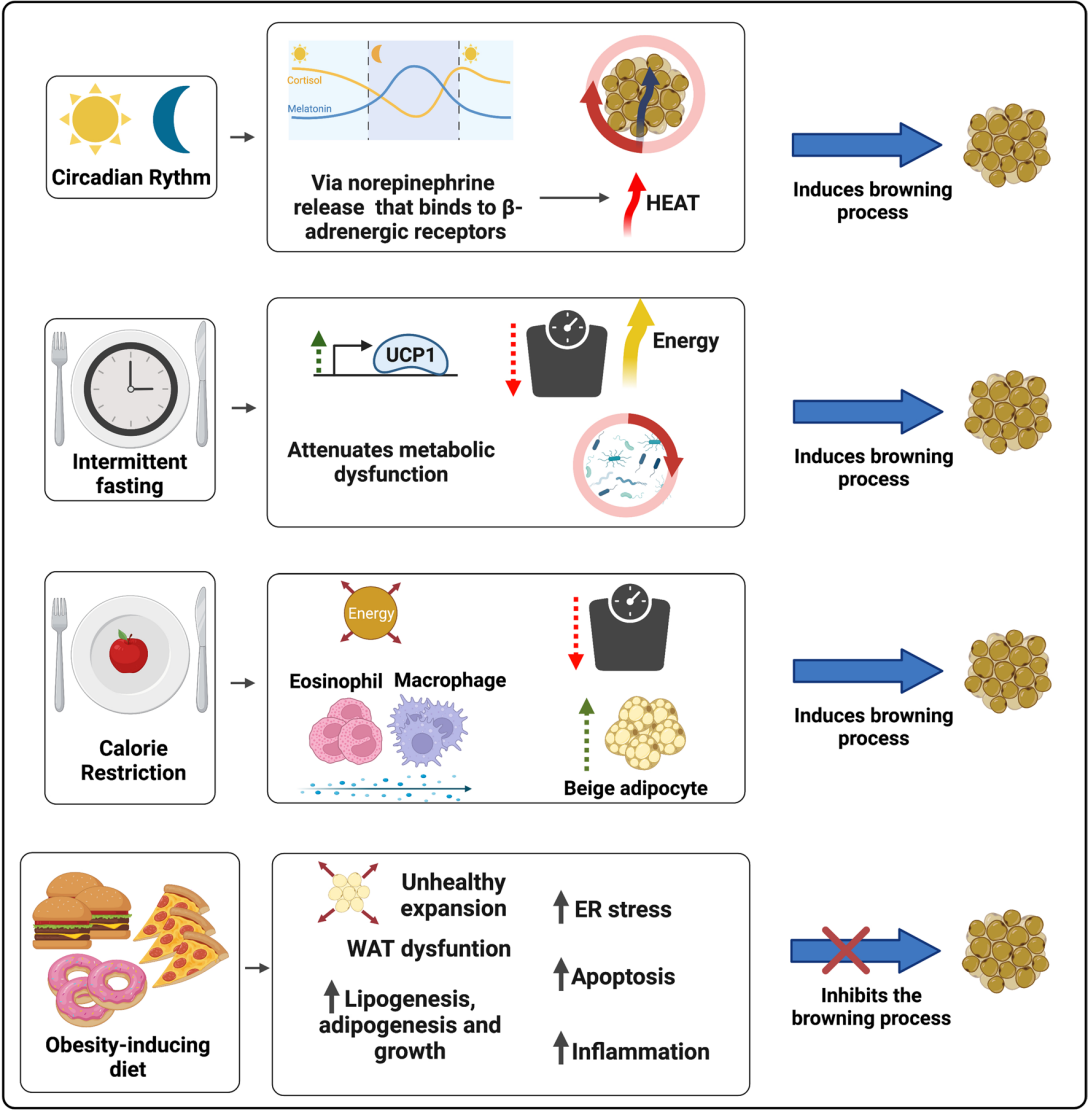
The impact of circadian rhythm and different diets on the WAT browning modulation. The secretion of melatonin, a circadian rhythm regulating neurohormone, is mediated by the release of Norepinephrine (NE), which binds to β-adrenergic receptors. Adrenergic activation is one of the main mechanisms of WAT browning induction and BAT activation. Intermittent fasting (IF) associates with weight reduction, improved metabolic status due to increased glycemic tolerance, decreased white adipocyte hypertrophy and AT inflammation, and augmented expression of thermogenic genes (such as UCP1) and recruitment of beige adipocytes. IF is also modulates the intestinal microbiome composition and diversity, a shift closely related to the induction of browning in the WAT. Caloric restriction (CR) is also associated with weight loss, promotes greater recruitment of beige adipocytes through the participation of M2 macrophage and eosinophil infiltration and in WAT. Finally, obesity-inducing diets correlate with increased lipid accumulation, WAT unhealthy expansion and dysregulation. Abnormal expansion of WAT promotes ER stress, greater induction of adipose cell apoptosis and inflammation through NF-κB transcription factor activation and increased pro-inflammatory cytokines secretion

The stress resistance provided by the IF practice places this regimen as a feasible dietary intervention against various devastating complex pathologies. Differently from CR, intermittent fasting (IF) does not influence the meal size, but decrease the number of meals in a given period [232]. The fasting state leads to a metabolic switch, which increases the usage of free fatty acid (FFA) as energy source in comparison to glucose. In addition, IF favors the synthesis of ketone bodies (KBs) by the liver, molecules that act as an energy source during nutrient deprivation and induce a plethora of beneficial effects on the organism by acting upon the muscle, liver, heart, brain, intestine and AT [235–237]. Moreover, during prolonged fasting periods, the levels of the bioenergetic sensors NADH, ATP, and acetyl-CoA decrease and the amounts of NAD+, AMP, CoA rise, molecules that act as epigenetic cofactors and lead to the activation of stress resistance mediators, as sirtuins (SIRTs), NRF2 (nuclear factor erythroid 2–related factor 2), and AMPK (AMP-activated protein kinase). IF also impacts positively on AT remodeling. A DIO animal model submitted to repetitive fasting cycles displayed increased glucose tolerance, and diminished adipocyte hypertrophy and tissue inflammation [238]. Mouse studies show that IF induces WAT mass decrease, elevation of AT UCP1 expression and thermogenic capacity [239, 240], and augmented beige pre-adipocytes recruitment to WAT [241–243] (Fig. 2).

An elegant study conducted by Li and colleagues informed that mice under IF cycles display an intestinal microbiome composition shift associated with increased levels of the fermentation products lactate and acetate. They also show that the modulation of the gut microbiota by IF is crucial for its browning effect, as microbiota-depleted mice present impaired IF-induced AT beiging and fecal microbiota transfer from these mice to antibiotics treated animals display increased browning of WAT [244] (Fig. 2). Unexpectedly, a human study conducted by von Schwartzenberg and colleagues showed that CR may diminish bacterial abundance, deeply change gut microbiome composition and diversity, impair nutrient absorption, and favor the outgrowth of the pathobiont (*Clostridioides difficile*). This diet also led to a decrease in bile acid (BA) levels [245].

BA, nonesterified fatty acids, are synthesized during the browning of WAT, a phenomena associated with the potentiation of the lipolytic machinery [246]. These fatty acids can not only activate UCP-1 allosterically, but also serve as fuel for oxidative phosphorylation and consequently heat generation in BAT [1]. Furthermore, in the liver they are used for the generation of acylcarnitines and VLDL which is used as source for thermogenesis [247]. Moreover, studies show that the increase in brite and brown adipocytes in WAT leads to an elevation in lipoprotein lipase (LPL) activity and subsequently an increase in circulating lipids available for BAT through intravascular hydrolysis of chylomicron triglycerides [248]. Consequently, these mechanisms result in the generation of cholesterol-enriched lipoprotein remnants, which upon activation of BAT accelerates the flow of cholesterol to the liver [249].

BA are steroid acids derived from dietary cholesterol catabolism. These acids are synthesized in the liver and act to aid digestion and absorption of fat in the intestine, in addition to playing an essential role in lipid metabolism. BA act in other tissues, such as AT, as signaling molecules through interaction with the nuclear Farnesoid X receptor (FXR) and the G protein-coupled membrane receptor (TGR5) [250]. Recent studies have shown that BA play a relevant role in BAT activation and increased thermogenesis in adipocytes. In rodents, the activation of BAT by BA is dependent on its interaction with the TGR5 receptor and expression of the enzyme type 2 iodothyronine deiodinase (DIO2). Additionally, experiments with oral supplementation of BA in humans indicated increased BAT activity in humans [251, 252]. Another experiment performed under thermoneutrality, demonstrated an improvement in glycemic metabolism and lipogenesis in the liver and fat accumulation in the TA and also induced an improvement in thermogenic parameters and mitigation of the impact of diet-induced obesity after feeding mice with HFD associated with BA [253].

Moreover, BAT activation also promotes liver protection. In a study performed with animals under alcohol-induced hepatic steatosis or liver injury, activation of the TGR5 receptor induced improvement of clinical condition. The increase in thermogenesis in BAT promotes an increase in lipid metabolism with lower availability of circulating FFA and, consequently, lower absorption of these molecules by the liver [254]. However, if on the one hand BAs have been shown to be effective in inducing browning, on the other hand the excess of these acids is capable of promoting an antagonistic effect, such as mitochondrial dysfunction and expression of genes associated with cellular senescence in adipose cells [255].

### Transcriptional regulation of WAT browning

ATs are embryologically distinct from other tissues and are formed according to specific stimuli during embryo development, including Bone morphogenetic proteins (BMPs), pleiotropic molecules that interact with type I and type II BMP receptors and influence embryogenesis [256, 257]. BMPs interact with type I and type II BMP receptors serine/threonine kinase activity and influence lineage determination [258]. Tang and colleagues showed that transfection of C3H10T1/2 cells with BMPs coped with phenotypes: while BMP2 associated with the osteogenic lineage, BMP4 led to adipogenic differentiation [259].Noteworthy, BMP4 overexpression was found to increase UCP1 and other beiging markers, as Hoxc9, Tbx1, and Tbx15 [260]. These different phenotypes induced by the BMPs, including the induced beige adipocytes, highlight how relevant transcriptional regulation is for determining the cells functions and characteristics. The main proteins that regulate gene expression are the transcriptional factors (TFs), DNA binding proteins that modulate gene transcription by interacting with the gene promoter or cis-regulatory elements, such as enhancers and silencers, and include PPAR proteins, PGC-1α, and PRDM16 [261, 262].

In addition to its roles in ATs development [263], PPARγ is a central TF for adipogenesis and lipid storage regulation, influences cell thermogenic capacity, and impacts lipid metabolism and insulin sensitivity [264]. This TF is expressed in elevated levels in ATs [265], and upon ligand binding PPARγ recruits different cofactor sets for controlling the expression of specific genes. PPARγ cooperates with the basic leucine-zipper factor C/EBPα and interacts with the majority of adipocyte-selective genes [266, 267]. The use of PPARγ full agonists is associated with improved insulin sensitivity and induces WAT browning, but can cause detrimental effects, such as undesirable weight gain and augmented visceral adiposity [264].

Although PPARγ is sufficient for converting white adipocytes into cells that display a brown-like phenotype in vitro [268], PPAR-α and PPAR-β/δ also influence the browning process. PPAR-β/δ agonist enhances beta-oxidation and improves glucose tolerance, key characteristics of the white-to-beige plasticity [269]. PPARα acts synergistically with PPARγ in inducing robust WAT browning in vivo [268], and is currently considered a prominent target for treating metabolic disorders [269].

A way that PPAR agonists provoke WAT browning is by stabilizing PRDM16 [270], a protein that activates a complete set of thermogenic genes in WAT [271]. PRDM16 is essential for browning particularly in scWAT, once its induction in visceral depots does not correlate with thermogenesis [272]. Mice lacking Prdm16 in scWAT are unable to induce browning within subcutaneous depots after stimuli [273]. Ectopic PRDM16 expression induce thermogenic genes in several cell types [274]. PRDM16 AT overexpression in rodents copes with augmented energy expenditure and DIO resistance [272] PRDM16 acts by binding to specific regulatory sequences in DNA and by interacting with other proteins [271], such as PGC-1α [275].

PGC-1α plays a key role in the adapting thermogenesis. First described in cold-induced adaptive thermogenesis analyses [276], this transcriptional coactivator participates in the regulation of a plethora of cellular functions, including mitochondrial biogenesis, oxidative phosphorylation, and gluconeogenesis [277, 278]. Once PGC-1α influences genes related to energy metabolism, it is expressed mostly in tissues that require an elevated amount of energy, like AT, liver, skeletal muscle, and brain [279]. Pgc1α is activated by the action of the cAMP-PKA-p38/MAPK signaling pathway and physically interacts with Nuclear Respiratory Factors 1 and 2 (NRF1 and NRF2) and co-activates PPARγ, PPARα, and ERRα/β/γ [280]. When overexpressed, Pgc1α induces mitochondrial biogenesis [281].

Another key regulator of the browning process is CIDEA. Initially described as a mitochondrial protein, CIDEA was further discovered to be associated with cell lipid droplets (LD) [282–284]. This molecule leads to the occurrence of browning by inhibiting the suppression of UCP1 gene expression mediated by liver-X receptors (LXRs) and increasing PPARγ binding strength to the UCP1 enhancer [285]. As detailed in this review, UCP1 is found in the inner mitochondrial membrane and acts by uncoupling the electron transport chain and oxidative phosphorylation, releasing energy as heat [1]. The existence of molecular markers for the browning process can be useful for investigating AT plasticity status and correlate with health and disease.

Zfp516 is a TF that directly binds to the UCP1 and PGC1α promoters and induce WAT browning upon cold exposure. Zfp516 overexpression copes with augmented multilocular lipid droplets (LDs) biogenesis and increased oxygen consumption and UCP1 levels [286]. HSF1 was described to cooperate with PGC1α in igWAT, favoring the induction of the thermogenic and mitochondrial gene programs, which leads to augmented energy consumption. HSF1-deficient mice are cold intolerant due to decreased β oxidation and UCP1 expression. HSF1 activation associates with scWAT browning, non-shivering thermogenesis and energy consumption [287, 288]. IRF4 also cooperates with PGC1α and was described to inhibit lipogenesis in adipocytes [289]. While IRF4 overexpression favors beiging in epididymal WAT, the absence of this factor is linked to diminished energy expenditure and increased risk to hypothermia [290]. The TF NFIA presents a crucial role in the initial steps of thermogenic gene regulation, as its increased levels in thermogenic adipocytes precedes PPARγ upregulation in these cells [291].

Members of “Early B-Cell Factor” (EBF) protein family play key roles in the regulation of thermogenesis. The TF EBF2 uncouples adipocyte mitochondrial respiration and is sufficient for WAT browning. Increased EBF2 levels in WAT leads to activation of the thermogenic program, favoring increased oxygen consumption and resistance for weight gain. EBF2-KO mice show impaired WAT browning and ablates the brown fat-specific characteristics of BAT [292–294]. EBF2 activity is regulated by the action of ZFP423, which binds to EBF2 and recruits NuRD (nucleosome remodeling deacetylase) corepressor complex to suppress EBF2 activity in thermogenic genes regulatory sequences [295]. Absence of ZFP423 associates with PPARγ binding to thermogenic gene enhancers, WAT browning and non-shivering thermogenesis [296]. The protein transducin-like enhancer of split 3 (TLE3) was first described by Villanueva and colleagues to increase PPARγ adipogenic activity [297]. Deletion of TLE3 copes with increased energy expenditure and mitochondrial oxidative metabolism in adipocytes, characteristics associated with the browning process [298] KLF11, TAF7L, ZBTB16, EWS, PLAC8, ERRα, ΕRRγ and other TFs that were described to promote WAT browning. In contrast, FOXO1, TWIST1, p107, LXRα, pRB, RIP140, REVERBα acting repressing AT beiging by impacting on the activity of EBF2, PRDM16, PGC1α and other activating TF [299–301].

In order the transcription to occur, chromatin needs to be accessible to polymerases and TFs. Remodeling enzymes, which are classified in covalent histone modifiers and ATP-dependent chromatin remodeling complexes, actively modify chromatin status in response to environmental cues [302]. Histone covalent modifiers are enzymes that chemically modify positively-charged amino acids (mainly lysine) present in these proteins. Histone acetyltransferases (HATs) and histone deacetylases (HDACs) catalyze the removal or insertion of acetyl group, respectively, influencing histone acetylation pattern [303]. Acetylation decreases histones’ positive charge, leading to histone-DNA looser interaction, chromatin decompaction and gene activation. These epigenetic elements classified into three major families based on the acetyl group transfer mechanisms: the CREB binding protein (CBP)/P300 family (CBP, P300), the GCN5-related N-acetyltransferases (GNAT) family (GCN5, PCAF, and Hat1), and the MYST family (MYST1, MYST2, TIP60) [304]. The HAT CBP was shown to inhibit the browning process, once it is key in white adipocyte differentiation [305]. Histone acetylation pattern has been described to present a critical role in adipocyte identity, as cold exposure leads beige adipocytes to show histone acetylation pattern associated with brown phenotype [306]. The HATs GCN5/PCAF were also shown to participate in the browning process [307].

HDACs catalyze the deacetylation of amino acid residues in histones, reactions favor gene repression. These enzymes are categorized into four groups, namely class I (HDAC1–3 and 8), class IIa (HDAC4, 5, 7, and 9), class IIb (HDAC6 and 10), class III (SIRT1–7), and class IV (HDAC11) [304]. HDAC1 expression is augmented in WAT and copes with decreased levels of proteins associated with non-shivering thermogenesis, as UCP1 and PPARγ [308]. HDAC3 levels also suppress WAT browning, as absence in WAT correlates with H3K27ac on enhancers of Ucp1 and Ppar*g*, signature associated with tissue improved oxidative capacity, mitochondrial biogenesis, and thermogenesis [309]. Experiments involving the deletion of HDAC9 shows that this enzyme also contributes for the metabolic dysfunction characteristic of HFD-fed rodents [310]. HDAC11 is another element that impairs WAT beiging, once its removal favors at thermogenesis in diet-induced obese (DIO) mice [311]. Other key HDAC is the SIRT family (SIRT1, SIRT2, SIRT3, SIRT5, and SIRT6). SIRT1 deletion in HFD-treated mice is associated with diminished amounts of PGC-1α, FGF21, and UCP1 in epididymal WAT (eWAT) [312].

Histones can also be post-translationally modified by histone methyltransferases (HMTs) and histone demethylases, which influence gene activation or suppression depending on the modified residue position and valency [313]. There are two categories of HMTs: lysine methyltransferases (KMTs) and arginine methyltransferases (RMTs). Rodent studies show that inactivation of the KMT MLL3 favors improved insulin sensitivity and augmented energy expenditure [314]. Absence of another KMT, the EHMT1, was shown to impair the thermogenic program in WAT [315]. Histone demethylases (HDM) catalyze histone demethylation processes and are also classified according to the characteristics of the modified amino acid. The KDM LSD1 expression, which was found to be induced by chronic cold exposure and β3-adrenergic stimulation, leads to increased mitochondrial activity in WAT by cooperating with NRF1 [316–318]. In addition, rodents presenting increased LSD1 levels are associated with igWAT browning in lean animals and with lower weight gain in the context of DIO [317] Also induced by β3-adrenergic stimulation, the KDM3A JMJD1A directly regulates ppar*a* and ucp1 genes and was found to be crucial for WAT browning [319]. Specific DNA sequences called CpG islands can also be methylated for gene transcription regulation by DNA methyltransferases (DNMTs), which switches off gene expression. Ten-eleven translocation (TET) family enzymes switch on the gene expression by demethylation [320].

Gene transcription can also be impacted by the action of ATP-dependent chromatin remodeling complexes, which, differently from the covalent histone modifiers, alter the interaction between the DNA and the nucleosome non-covalently using ATP hydrolysis as an energy source. The chromatin remodelers are categorized in INO80, CHD, ISWI, and SWI/SNF [321]. The mammalian SWI/SNF (mSWI/SNF or BAF) complex can act with either Brahma homolog (BRM) or BRM-related gene 1 (BRG1) ATPases [322]. Abe and colleagues suggested that BRG1 is necessary for thermogenesis induction [319].

Other key actors in gene transcription regulation are the microRNAs (MiR), small non-coding regulatory ribonucleic acids (maximum 200 nucleotides) that influence gene expression post transcriptionally. These elements regulate gene expression by several mechanisms, including binding to mRNA strands to repress protein translation and to favor decapping, deadenylation and ultimately degradation of target mRNA in P-bodies, and direct translation inhibition. However, many studies suggest that miRNAs are also capable of activating transcription protein translation, fact that highlights miRNAs as central participants in the fine regulation of gene expression in response to specific stimuli [323]. Raymond and others described that miR-32 inhibition copes with impaired WAT browning [324]. MiR-181, which is induced by tryptophan‐derived metabolites produced by the intestinal microbiota, was also connected with WAT physiology, once it promotes energy expenditure and insulin sensitivity in rodent models and its removal may cope with the development of obesity in these animals [325]. Another miRNA that influences WAT homeostasis is miR-26, which deletion leads to WAT enlargement and overexpression inhibits the progression of obesity in the DIO in mice [326]. miR-196a, miR-455, miR-30b/c are other examples of miRNAs that are induced by browning inducers and promote this WAT plasticity process [327, 328].

**Fig. 3:**
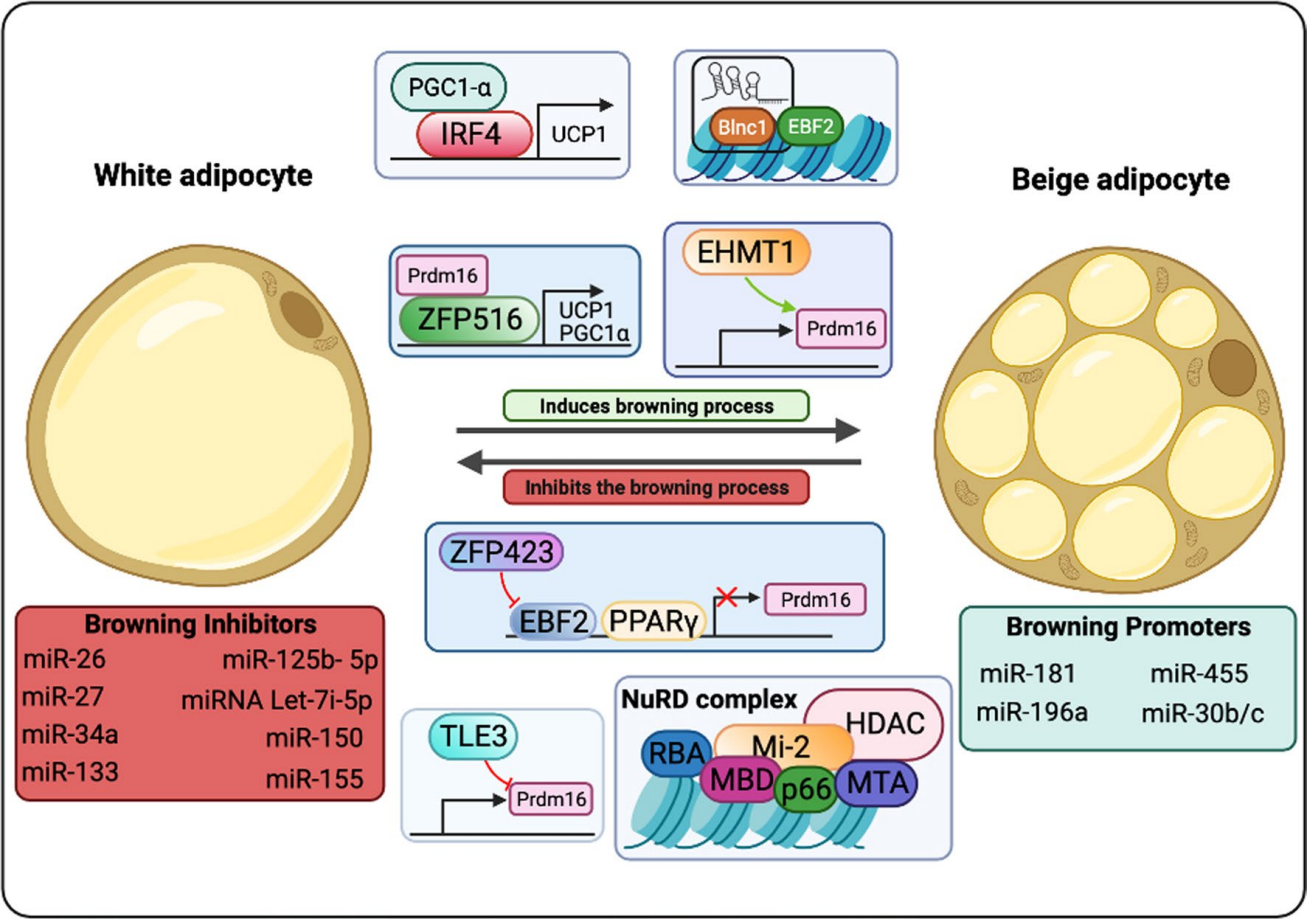
WAT browning transcriptional regulation. The transcriptional regulation of the WAT beiging process involves the action of a plethora of specific proteins and nucleic acids. This orchestra of trans-acting factors modulates genes associated with oxidative capacity, mitochondrial biogenesis and non-shivering thermogenesis. While the action of some factors, such as PGC-1α, IRF4, PRDM16, ZFP516, EHMT1 and RNAs, copes with WAT browning, TLE3, ZFP423, and the NuRD complex, another set of RNAs e others inhibit this process, favoring adipogenesis and white adipocyte differentiation

In contrast, miR-27, miR-133, and miR-150 inhibit TFs associated with the browning process [61, 63, 329]. Fu and colleagues showed that miR-34a inhibits WAT beiging via FGF21 [330], miRNA-155, miRNA Let-7i-5p and miR-125b- 5p are molecules that impair WAT browning process, which inhibition promotes beiging [331–333] (Fig. 3). Other small non-coding RNAs (sncRNAs) impact on gene expression and are extensively reviewed elsewhere.

Non-coding RNAs that present average length longer than 200 nucleotides are called LncRNAs [302, 334] These nucleic acids have been described to influence gene transcription through several mechanisms, including the induction of more efficient protein translation by binding to an internal ribosome entry site (IRES), increase of mRNAs stability, polyubiquitination process inhibition thus increasing protein stability, binding to specific proteins in the cytoplasm, and acting as a sponge sequestering miRNAs [335–339]**.** Zhao and collaborators identified lncRNA 1 (Blnc1) as a driver for beige adipocyte differentiation and WAT browning [293].

All the regulatory elements discussed here cooperate for generating a phenotype in response to environmental stimuli, such as physical exercising, intermittent fasting, calorie restriction, thyroid hormones, microbiota-associated metabolites, thyroid hormones and others [299] The lncRNA Blnc1 forms a Blnc1/hnRNP-U/EBF2 ribonucleoprotein complex and is required for EBF2 transcriptional activity [292, 340]. The TF ZBTB7B also interacts with this lncRNA through the hnRNP-U/Blnc1 for beige adipocyte differentiation and thermogenesis [341]. LncRNAs also interact with other TFs, such as PGC-1α, ZFP516, and PRDM16 for full transcription [274, 280, 286]. PRDM16, a coregulator of PPAR, cooperates with EHMT1 for WAT browning induction. As EHMT1, other covalent histone modifiers form complexes with ATP-dependent chromatin-remodelers [342]. The chromatin-remodelers BRG1 and the BAF also interact with the TF EBF2 for gene transcription [292, 293]. PGC1α favors increased transcription by recruiting HATs, as CBP/p300 and GCN5 [343].

Noteworthy, AT functional and morphological plasticity is represented by the extreme dynamic beige adipocyte chromatin state. Beige adipocytes show a chromatin signature similar to the pattern presented by brown adipocytes after cold exposure and display a chromatin signature associated with white adipocytes after re-warming to 30 °C. However, if the beige adipocytes were cold-induced, these cells displayed epigenetic marks that favored rapid thermogenic genes expression upon β-adrenergic stimulation, even after temperature rise, suggesting the occurrence of epigenomic memory [306, 344].

### WAT browning, health impact and applications

For the past decades, the browning process has been deeply explored for managing both metabolic syndromes, and hypermetabolic diseases. Proposing treatment, the cold was the pioneer mechanism associated with a great elicitation of this event in mice. However, the applicability and outcomes of this approach in humans are far from exciting. Technological advances provide efficient mechanisms that can be employed to improve a biological system. Recent studies applied bioengineers to achieve the improvement of browning in adipose tissue. The injection of M2 AT macrophages (ATMs) from transgenic mwRIP140KD mice, a specific knockdown of RIP140 that is related to activation of M1 polarization, in HFD-fed obese wild type mice could recover the disruption induced by obesity through browning induction [345]. WAT-derived stem cells (ASCs) from humans and rats could be differentiated into BAT under browning conditions in three-dimensional (3D) polyethylene glycol (PEG) hydrogel [346].

In addition to bioengineering, a field that has been explored is the transplantation or re-implantation of BAT, also termed ex-BAT, to increase this endogenous tissue. The harvested scWAT is differentiated into brite and then is re-implanted in the same area promoting the local increase of BAT, displaying great outcomes [347]. Aiming for a less invasive procedure, the use of a microneedle patch to address the browning agents directly to the scWAT for inducing browning of this tissue is another strategy investigated presently [348]. These strategies aim to promote the increase of endogenous BAT, as well as its activity with an applicable method, reducing the degree of invasiveness and risks of rejection by the body.

Several studies have targeted increasing BAT mass and activity, but when the aim is inhibiting the browning process to avoid a worsening prognosis, what is proposed? Expanding knowledge of the pathways involved is the main way of inhibiting that event. It is well established the role of parathyroid hormone (PTH) in the elicitation of browning. In the tumor context, it was identified as a peptide-derived tumor, parathyroid hormone-related protein (PTHrP) that favors browning and cachexia [16]. Blocking PTHR specifically in ATs inhibits browning and wasting of this tissue, as well preserves muscle integrity, and ameliorates muscle-related strength, protecting mice from cachexia [349].

Currently, some pharmacological approaches have displayed alternative and prominent ways to restrain browning. Metformin demonstrated to be efficient to prevent the browning of the scWAT, as well as the inhibition of mevalonate pathways by the use of Statin or Fluvastatin, which implicates in the disruption of browning [350, 351]. However, further studies should be conducted to promote a broader and more detailed debate on the topic.

Given the data presented, it is evident that the benefits and dangers associated with activation or inhibition of the browning process are closely linked to the type of disorders displayed in the individual's body at the metabolic, cellular, or physiological level. Taking as criteria patients with metabolic syndromes and obesity, browning process emerges as a promising therapeutic approach, mainly due to its several benefits, such as improvement of clinical conditions associated with lower side effects, and the multi-stimulatory character, which allows numerous safe and feasible ways of induction. In contrast, the ways and means of inhibiting browning to prevent the development of comorbidities in cases of chronic or acute hypermetabolism are still poorly explored. In this way, browning process needs to be deeply explored and can be used as a key factor in different therapeutical approaches in health and diseases. Emerging insights into the metabolic and immunological role of browning of the white adipose tissue are also discussed, along with the developments that can be expected from these promising targets for therapy of metabolic and chronic disease in the forthcoming future.

### Mouse and human AT research

A major discussion pointed by the scientific community about the investigation of AT in mice is how much the results obtained in the animal model would reproduce the anatomorphophysiologies of these tissues in humans, since, unlike humans, mice have a significant amount of BAT during embryonic and adult, as well as they differ in several other factors, such as expression of molecular markers, activation profile and location. Human BAT was discovered to be more similar to beige compared to classical BAT markers [352, 353]. Another relevant point concerns the fact that the BAT deposits most used for research purposes are the BAT located in the interscapular region (iBAT), while in humans there is a greater abundance of this tissue in the clavicular and neck regions, presenting compositional differences that represent obstacles in the overlapping of scientific findings. In this sense, Mo and colleagues identified an analogous deposit of BAT in mouse embryos, which is maintained during adulthood, and in humans called supraclavicular BAT (scBAT). The scBAT shows similarities to scBAT in humans in terms of location, morphology and thermogenic capacity [354].

Another alternative developed in order to attenuate the anatomical, physiological and molecular differences between the organisms was to submit mice under conditions called “physiologically humanized” in which middle-aged animals under thermoneutrality (30˚C) and diets aligned with the dietary pattern of human beings are used. humans. In which the authors observed a greater similarity between several cellular and molecular parameters between the BAT of humans and mice [355]. The technique generated controversies that were discussed by Kajimura and Spiegelman and replicated by the authors of the original article (356, 357).

## Conclusions and perspectives

Current research place WAT browning as an extremely dynamic process that is influenced by several factors**,** including temperature, physical exercising, thyroid hormones, circardian rhythm, food components and dietary regimens. The participation of AT plasticity on the organism metabolic health and inflammatory status spot this process as a promising therapeutic target for decreasing the risk associated with many chronic diseases. Further efforts in investigating AT plasticity must alleviate the burden of these devastating life-style associated pathologies.

## Data Availability

Not applicable.

## Abbreviations

D2: 5′-Deiodinase type 2
Aa: Amino acids
AC: Adenylyl cyclase
AMPK: AMP-activated protein kinase
ANP: Atrial natriuretic peptide
ASC: Adipose stromal cells
AT: Adipose tissue
ATF: Activating transcription factor
ATMs: Adipose tissue macrophages
ATP: Adenosine triphosphate
BAT: Brown adipose tissue
BBB: Blood–brain barrier
BMP: Bone morphogenetic proteins
BMR: Basal metabolic rate
BNP: Brain natriuretic peptide
BRG1: BRM-related gene 1
BRM: Brahma homolog
C/EBP: CCAAT-enhancer-binding protein
Ca2+: Calcium ion
cAMP: Cyclic adenosine monophosphate
CBP: CREB binding protein
ChREBP: Response element-binding protein
CIDEA: Cell death-inducing DNA fragmentation factor-like effector A
CLAs: Conjugated linoleic acids
CNS: Central nervous systems
CPT1B: Carnitine palmitoyltransferase 1B
CR: Calorie restriction
CREB: CAMP response element-binding protein
CREBH: Cyclic AMP response element-binding protein H
DHA: Docosahexaenoic acid
DIO: Diet-induced obese
DIO2: 2 Iodothyronine deiodinase
DIT: Diet-induced thermogenesis
DNMT: DNA methyltransferases
eAT: Epicardial AT
EBF: Early B-cell factor
eFGF: Endocrine FGF
eIF: Eukaryotic initiation factor
EPA: Eicosapentaenoic acid
eWAT: Epididymal WAT
FAs: Fatty acids
FFAs: Free fatty acids
FGF: Fibroblast growth factor family
FGFR: Fibroblast growth factor tyrosine kinase receptor
FNDC5: Fibronectin domain-containing protein 5
FRS2α: FGFR substrate 2α
FXR: Farnesoid X receptor
GCN2: Non-derepressible 2
GLUT: Glucose transporter
GNAT: GCN5-related N-acetyltransferases
HAT: Histone acetyltransferase
HDAC: Histone deacetylase
HDM: Histone demethylase
HFD: High-fat diet
HMT: Histone methytransferases
iBAT: Interscapular BAT
IF: Intermittent fasting
IL: Interleukin
IRES: Internal ribosome entry site
K+: Potassium
KLB: Beta-Klotho
KMY: Lysine methyltransferases
LCFA: Long-chain fatty acids
LD: Lipid droplet
lnc-RNA: Long non-coding RNA
LPS: Lipopolysaccharide
LXR: Liver X receptor
MACF: Mitochondrial anion carrier family
MCT8: Membrane proteins monocarboxylate transporter 8
MiR: MicroRNA
miRs: MicroRNAs
mRNA: Messenger RNA
Mstn: Myostatin
My f5: Myogenic factor 5
Na+: Sodium
NAFLD: Nonalcoholic fatty liver disease
ncRNAs: Non-coding RNAs
NE: Norepinephrine
NFR2: Nuclear factor erythroid-related factor 2
NRF1: Nuclear respiratory factor 1
NRF2: Nuclear respiratory factor 2
NuRD: Nucleosome remodeling deacetylase
OATP1C: Organic anion transporter family member 1C1 9
OPE: Onion peel extract
p38 MAPK: Mitogen-activated protein kinase
PD: Pyruvate dehydrogenase
PEG: Polyethylene glycol
PGC1-α: Peroxisome proliferator-activated receptor-gamma coactivator 1 α
PKA: Protein kinase A
POMC: Proopiomelanocortin
PPAR: Peroxisome proliferator-activated receptor
PRDM16: PR domain containing 16
PTH: Parathyroid hormone
PTHR: Parathyroid hormone receptor
PTHrP: Parathyroid hormone-related protein
PUFAs: Polyunsaturated fatty acids
RA: Retinoic acid
RAR: Retinoic acid receptor
RIP140: Receptor-interacting protein 140
RMT: Arginine methyltransferases
RORα: Retinoic acid receptor-related orphan receptor α
rWAT: Retroperitoneal white adipose tissue
RXR: Retinoid X receptor
scBAT: Supraclavicular BAT
SCN: Suprachiasmatic nucleus
scWAT: Subcutaneous WAT
SIRT: Sirtuin
snc-RNA: Small non-coding RNA
SNS: Sympathetic nervous system
T2D: Type 2 diabetes
T3: Triiodothyronine
T4: Thyroxine
TBX1: T-box transcription factor TBX1
TET: Ten-eleven translocation
TF: Transcription factor
TGF-β: Transforming growth fator beta
TGR5: G protein-coupled membrane bile acid receptor
TH: Thyroid Hormone
Th2: T help 2 cells
TLE3: Transducin-like enhancer of split 3
TREs: Thyroid-hormone responsive elements
TRPM8: Transient receptor potential cation channel subfamily M member 8
TRPV1: Transient receptor potential vanilloid 1
TRs: Thyroid hormone nuclear receptors
TSH: Thyroid stimulating hormone
TSH-R: Thyroid stimulating hormone receptor
UCP1: Uncoupling protein 1
vWAT: Visceral WAT
WAT: White adipose tissue
WT: Wild type
ZAG: Zinc-α2-glycoprotein
β-3AR: β-3 Adrenergic receptor
